# Harnessing mmWave signals and machine learning for noninvasive taxonomic classification of insects

**DOI:** 10.1093/pnasnexus/pgag096

**Published:** 2026-04-28

**Authors:** Linta Antony, Cian White, Nicola Marchetti, Ian Donohue, Jane Catherine Stout, Adam Narbudowicz

**Affiliations:** School of Engineering, Trinity College Dublin, Dublin D02 PN40, Ireland; School of Natural Sciences, Trinity College Dublin, Dublin D02 PN40, Ireland; School of Engineering, Trinity College Dublin, Dublin D02 PN40, Ireland; School of Natural Sciences, Trinity College Dublin, Dublin D02 PN40, Ireland; School of Natural Sciences, Trinity College Dublin, Dublin D02 PN40, Ireland; Department of Space Research and Space Technology, Technical University of Denmark, Kgs. Lyngby 2800, Denmark

**Keywords:** insect biodiversity, species recognition, micro-Doppler signatures, mmWave radar, hierarchical classification

## Abstract

Monitoring insect biodiversity at sufficiently high resolutions in space and time is crucial to underpin robust and responsive management of terrestrial ecosystems. This study presents a novel approach for taxonomic classification of individual pollinating insects using millimeter-wave (mmWave) signal technology and a hierarchical machine learning (ML) framework. Although ML-based species identification has been proposed for image systems, its application to monitoring and classification of insects remains limited, due to high susceptibility to poor image quality and varying light conditions. The potential of mmWave systems to exploit ML for insect species recognition remains largely unexplored, however. mmWave radar offers access to biomechanical characteristics that are not visible to the human eye or cameras. These characteristics are encoded in the harmonic patterns generated by insect wingbeats and reflected in the radar signal. Here, we systematically explore signal features associated with wing flapping and employ SHapley Additive exPlanations analysis to identify the most discriminative features contributing to classification performance. A hierarchical ML model was developed and achieved an accuracy of 85% in classifying five key pollinator species. Given the growing integration of mmWave systems in communication infrastructure, this method offers a scalable, cost-effective, and contactless solution for high-resolution monitoring of insect biodiversity.

Significance Statement Here, we propose a noninvasive and low-cost approach using millimeter-wave (mmWave) signal technology for the taxonomic classification of insects based on wing-flapping patterns. By capturing biomechanical information encoded in mmWave reflections due to insect wing flaps, the proposed hierarchical machine learning framework enables effective species identification. Additionally, the integration of explainability techniques enhances understanding of the critical signal features involved. Given the increasing deployment of mmWave technology for joint communication and sensing applications, this method holds significant promise for scalable and sustainable monitoring of insect biodiversity.

## Introduction

Stemming rapid global biodiversity decline is critical to ensure the sustainability of human civilization (1). The capacity for robust and responsive management of ecosystems and the conservation of the biodiversity they support is, however, typically limited by data at excessively coarse resolutions in both space and time (2). This is particularly the case for pollinating insects, as traditional taxonomic identification of pollinators is labor-intensive, requires highly trained personnel, and usually involves lethal sampling (3). Fortunately, advanced machine learning (ML) offers the promise to expedite some of the routine classification tasks.

Currently, most works propose the ML classification of insects based on visual image processing (4, 5). This is a natural extension of the success of ML-based image recognition and classification observed in broader consumer electronics and smart applications. Early approaches used yellow sticky traps along with image processing and ML algorithms to leverage distinctive morphological features such as the shape, color, and texture of insects (6). The advent of deep learning (DL), particularly convolutional neural networks (CNNs), has revolutionized the field, enabling automatic feature extraction and learning from raw image data (7). For instance, to detect the most damaging pests in cotton fields, Alves et al. (8) introduced a dataset with images from these fields, comprising 15 categories and 100 images, using a CNN model with ResNet34 architecture to achieve an impressive classification accuracy of 97.8%. Similarly, Kasinathan et al. (9) employed a publicly available dataset of 1,387 images across 24 classes against complex backgrounds. However, despite the successful application of ML for image processing, its use for monitoring and classification of insects in the field remains limited. A major limitation of image-based insect monitoring is the difficulty of obtaining usable images, as many insects are highly mobile and often fly away when approached, combined with a high susceptibility to poor image quality caused by variable lighting conditions, inclement weather, and complex or cluttered backgrounds.

A complementary alternative is the use of radar systems (10). Radar has been used for decades in entomology to study migratory insects flying in large numbers at high altitudes (11, 12). These systems are typically designed for wide-area, long-range monitoring using weather or surveillance radar infrastructures. Recent studies have demonstrated that modern signal processing and ML techniques can significantly enhance the ecological utility of migration-focused radar systems. For example, deep learning and semisupervised frameworks have been applied to weather radar data to distinguish insect, bird, and precipitation echoes and to characterize large-scale migration dynamics (13, 14). Multiscale feature fusion networks and ensemble learning approaches using electromagnetic scattering parameters have further improved classification accuracy for migratory insects and birds (15, 16). While these advances highlight the growing role of ML in aeroecological radar, they remain primarily focused on large-scale migration monitoring rather than, near-ground sensing of individual insects such as pollinators visiting flowers.

Insect wing flapping generates distinctive micro-Doppler signatures, providing valuable insights for radar-based analysis and identification (17). In Ref. (18), wingbeat frequency was extracted from micro-Doppler signatures using insects tethered to polystyrene foam to enable controlled laboratory recording of wingbeat signals, rather than to capture natural free-flight behavior. Diyap et al. (19) used a 94 GHz radar to capture micro-Doppler signatures of mosquitoes and honeybees flying in controlled paths, clearly differentiating the two insect types based on wingbeat frequencies extracted using a cepstrogram-based method. Antony et al. (17) show the feasibility of differentiating *Apis mellifera* (honeybee), *Bombus terrestris* (bumblebee), and *Vespula vulgaris* (wasp) based on the wingbeat frequency extracted using pitch estimation filter (PEF) from the millimeter-wave (mmWave) communication signals. Aldabashi et al. (20) demonstrated a 5.8 GHz CW Doppler radar integrated with ML for automated honeybee hive surveillance, achieving behavioral classification by leveraging Doppler signatures at hive entrances.

In parallel with radar-based approaches, a number of studies have explored optical and photonic methods for insect detection and classification, often leveraging wingbeat-induced signals as key features. For instance, (21) emphasized that micro-Doppler–like optical effects, such as periodic modulations in backscattered light due to wingbeats, can serve as an optical analog to radar micro-Doppler phenomena. In particular, the presence of spectral harmonics in insect wingbeat signals was shown to carry valuable taxonomic information. Meanwhile, (22) demonstrated that the spectral fringes produced by the thin wing membranes of clear-winged insects can be a useful feature for remote insect identification. However, many existing approaches in radar entomology focus primarily on basic parameters such as wingbeat frequency extracted from micro-Doppler signatures generated by wing flapping, and rarely take full advantage of the harmonic content of these signals or the capabilities of modern ML for classifying closely related insect species.

**Figure 1:**
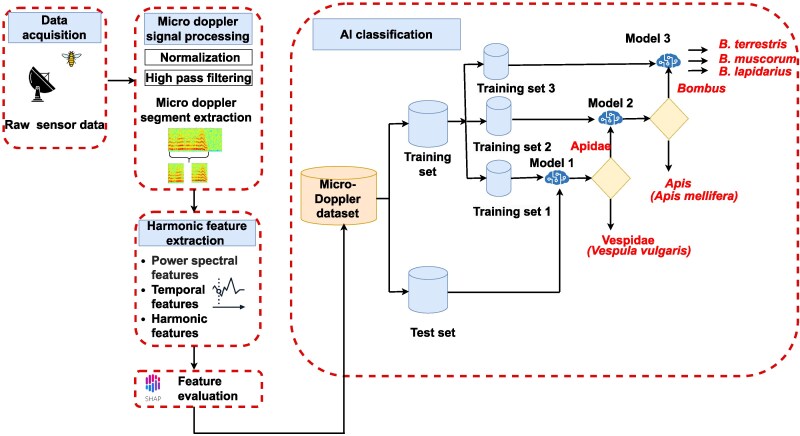
Workflow of the proposed mmWave micro-Doppler insect classification. The data acquisition process involves recording signals reflected from insects after transmitting a single-frequency unmodulated signal toward them. The signal segments containing wing-flapping information are filtered through a preprocessing step to isolate relevant data. From the filtered micro-Doppler segments, key features related to wing-flapping patterns are extracted. Feature importance is then assessed using SHAP, and a micro-Doppler dataset is created for different insect species. The dataset is split into training and testing data based on subject IDs, ensuring that no data from individuals in the test set is present in the training set. A three-level hierarchical classification model is then employed to perform taxonomic classification, encompassing classification to family, genus, and species levels.

Here, we propose a radically new approach (Fig. 1). Rather than focusing on morphological features that can be challenging for radar to detect, we exploit the harmonic content generated by the micro-Doppler effect of an insect beating its wings. This approach goes beyond relying solely on wing beat frequency, as it incorporates all relevant features that depict the micro-Doppler signatures that arise from wing flapping. Moreover, this method does not restrict natural wing movement. The analysis for each insect includes the full spectrum of micro-Doppler harmonics, from which key parameters, such as fundamental frequency, energy distributions, cepstral coefficients, and others, are extracted and used for training a three-level hierarchical ML architecture to categorize the signals taxonomically. By integrating explainable ML techniques, such as SHapley Additive exPlanations (SHAP), insight is provided into which signal features are most critical for differentiating species.

We use a hierarchical ML framework in which the first-level model is trained to predict the taxonomic family of the insect, the second-level model predicts the genus, and the third-level model predicts the species. We verify our method experimentally using a simple unmodulated Ka-band continuous wave signal that is back-reflected from the insect in a standard indoor setting with background scattering noise (ie due to laboratory environment with multiple reflective objects present, rather than in an anechoic chamber, resulting in realistic multipath and background clutter). We also analyze and quantify how both the distance of the target from the antenna and the duration of wing flapping affect the accuracy of the proposed classification. We find that mmWave signals offer real potential to provide cost-effective, rapid, and noninvasive identification and monitoring of pollinating insects at high resolutions in both time and space.

## Methodology

### mmWave signal recording

The research involves small number of invertebrate insects, which were released into their natural environment upon collecting the data. The research was approved by the ethical committee of School of Natural Sciences, Trinity College Dublin. Live insects belonging to the Order Hymenoptera were collected from the Trinity College Dublin campus and placed individually in small cylindrical plastic containers with a diameter of 4 cm and a height of 5 cm. Each container was placed on a plastic support on top of mmWave antennas (Broadband Horn Antenna BBHA 9170). A Rhode & Schwarz Vector Network Analyzer (VNA) was used to generate a single frequency continuous wave signal at 30 GHz, and the reflected signal (amplitude and phase) was recorded over the duration of 60 s. The VNA captured 60,001 samples during each 60 s measurement. The experiment was videotaped to establish the reference ground truth. Ground truth data were captured to provide a coarse temporal reference to identify periods of flight. No portion of the video recording was used for analysis, filtering, or identification. Those were based solely on radar data itself.

Data collection and experiments were carried out from May to November 2023. Since the insects were free to move during the observations, they displayed a variety of behaviors, including flying, wing flapping, and idleness, as well as engaging in distinct patterns of movement such as vertical and circular navigation within the containers. Measurements were specifically taken from subjects that exhibited wing flapping at least once, which was the primary focus of our analysis. Measurements were taken from a number of subjects from each species. However, only a subset exhibited the wing flapping required for subsequent analysis; the number in this subset is given in Table 1. Even in cases where wing flapping occurred alongside other movements, such as vertical or circular motions, these could be distinguished and filtered out in postprocessing, allowing for a focused analysis of wing flapping.

**Table 1:** Numbers of live insect subjects and flight segments of various durations used for training ML classification models.

Class	Subjects	2 s	1 s	0.8 s	0.6 s	0.4 s	0.2 s	0.1 s
*Apis mellifera*	11	1,244	2,416	3,002	3,950	5,860	11,491	22,320
*Bombus lapidarius*	4	1,168	2,122	2,620	3,358	4,815	9,085	17,335
*Bombus terrestris*	6	1,070	2,416	2,626	3,434	5,116	10,073	19,742
*Bombus muscorum*	5	680	1,372	1,716	2,253	3,334	6,572	12,925
*Vespula vulgaris*	7	931	1,755	2,106	2,729	3,995	7,772	14,756

### Problem formulation

**Figure 2:**
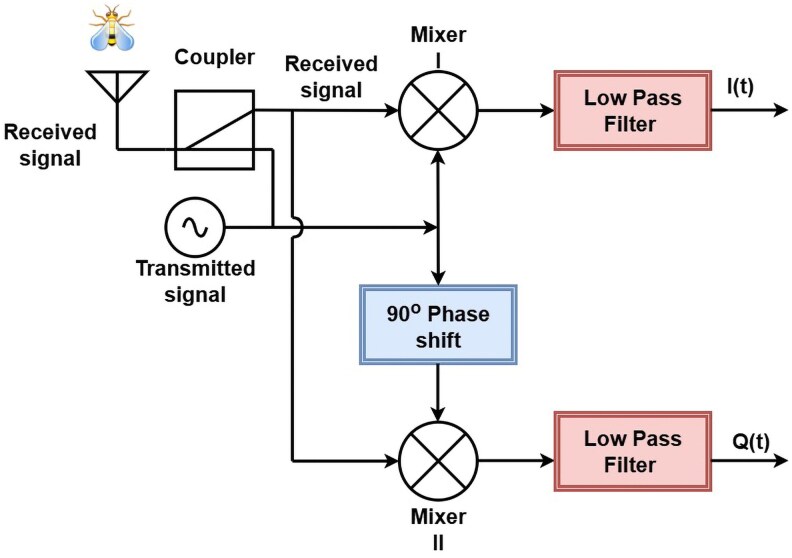
Structure of the transceiver.

A transmitter generates an unmodulated a single-frequency mmWave signal at 30 GHz and transmits it via an antenna. The antenna receives the backscattered signal from the target, which contains motion-induced modulations caused by insect wing movements. The reflected signal is then received by the same antenna and down-converted by mixing with the initially generated signal, thus extracting Doppler frequency (23). The structure of this transceiver is depicted in Fig. 2. The received signal is described by the expression $A\sin\;(\omega_{0}t+\phi )$, where $\omega_{0}=2\pi f_{0}$ is the angular reference frequency of the signal and *ϕ* the phase shift on a signal resulting from target motion. The down-converter performs quadrature demodulation by mixing the received signal with the transmitted signal using an IQ mixer. This process produces two orthogonal components: the in-phase (I) component, which is aligned in phase with the transmitted signal, and the quadrature (Q) component, which is $90^{\circ }$ out of phase. Together, the I and Q signals constitute a complex-valued representation of the received waveform, preserving both amplitude and phase information. This representation enables the extraction of micro-Doppler signatures associated with insect motion.

**Figure 3:**
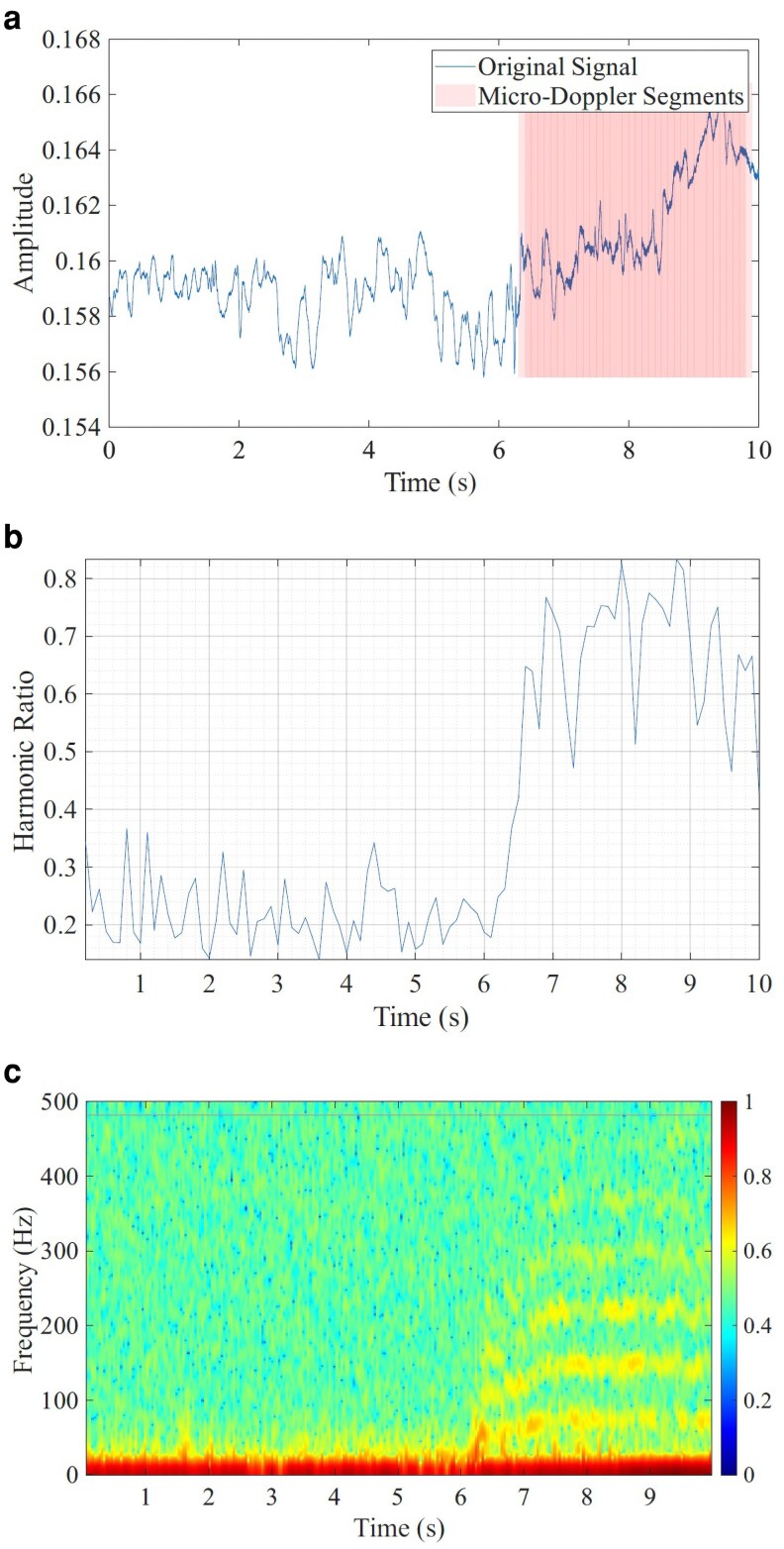
An example of micro-Doppler segment extraction from the radar signature of the common wasp *Vespula vulgaris*. The individual in the recording is initially stationary and commences flight after ∼6 s, as confirmed by the corresponding video footage. a) Raw recorded signal, where the time interval containing micro-Doppler activity is indicated and aligned with the harmonic ratio time series and spectrogram shown in (c). b) Harmonic ratio time series computed from the high-pass filtered mmWave signal (50 Hz cutoff). The harmonic ratio is a scalar harmonicity measure derived from the normalized autocorrelation: larger values indicate that a greater fraction of the signal energy is organized into a periodic (harmonic) structure. During the initial 6 s, when the insect is stationary, the harmonic ratio remains below 0.4. As the insect begins to flap its wings, the harmonic ratio increases significantly, indicating the emergence of periodic micro-Doppler features associated with wing motion. c) Short-time Fourier transform spectrogram of the unfiltered radar signal, showing micro-Doppler patterns as parallel horizontal bands between ∼6 and 10 s.

**Figure 4:**
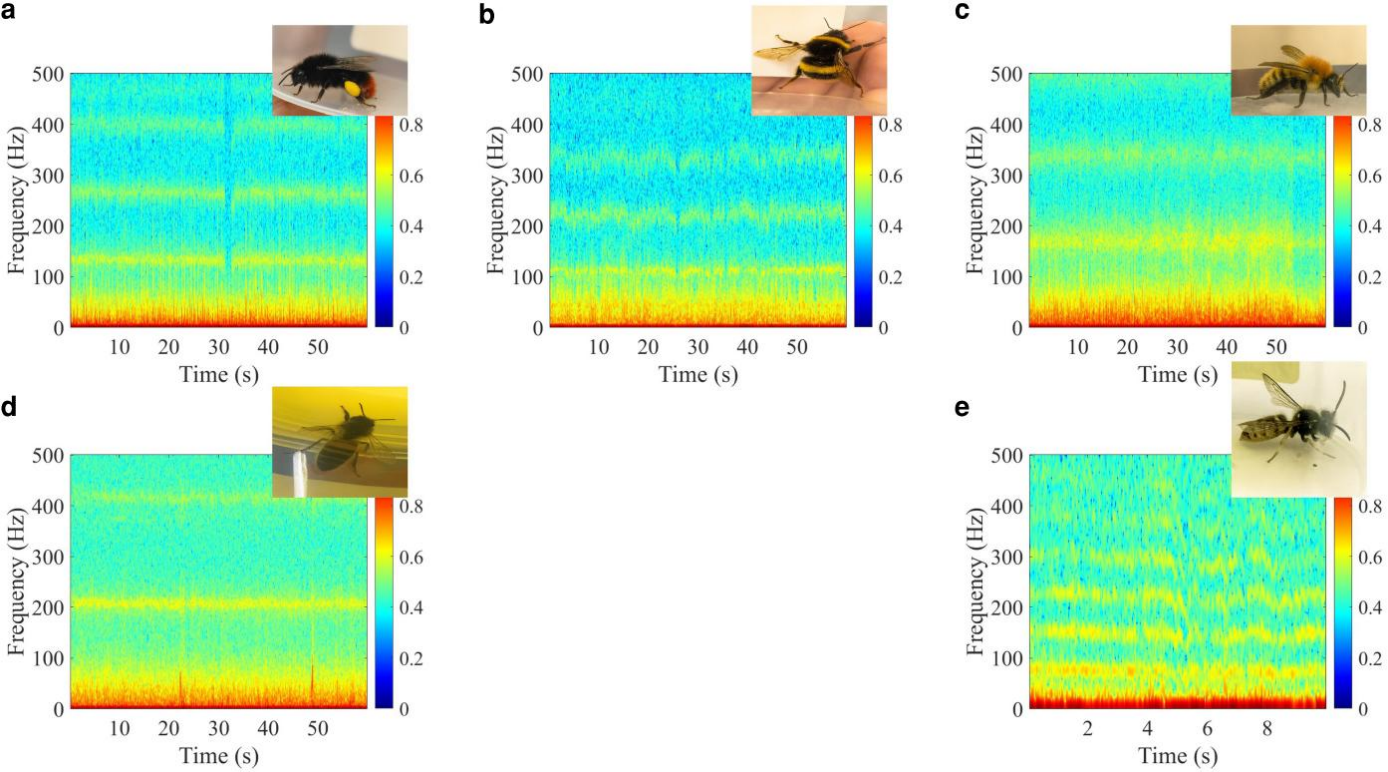
Exemplary reflected micro-Doppler spectrograms from wing flapping of five different pollinating insect species, computed from the unfiltered radar signals to illustrate the full micro-Doppler structure, including low-frequency components. a) *Bombus lapidarius*, b) *Bombus terrestris*, c) *Bombus muscorum*, d) *Apis mellifera*, and e) *Vespula vulgaris*. The reflected signals were recorded using a 30 GHz continuous wave signal. While differences among some species can be observed visually, a trained ML algorithm can recognize differences that are less apparent on visual inspection.

For the generic case where an insect is flying in front of the antenna, the complex down-converted micro-Doppler signal can be expressed as a combination of three components:


(1) $$A(t)e^{\;j\phi (t)}=s_{\mathrm{noise}}(t)+s_{\mathrm{body}\;\mathrm{movement}}(t)+s_{\mathrm{wing}\;\mathrm{beat}}(t)$$

where $s_{\mathrm{noise}}(t)$ corresponds to noise, including multiplicative background noise from all noninsect reflective objects; $s_{\mathrm{body}\;\mathrm{movement}}(t)$ is the Doppler signal originating from the movements of the insect due to its flight pattern; and $s_{\mathrm{wing}\;\mathrm{beat}}(t)$ is the micro-Doppler periodic reflection due to wing movement. Since the latter component is periodic, that is, modulated by the repeated wing movement, it can be expressed as the sum of harmonic signals with the lowest frequency corresponding to the frequency $f_{w}$ at which the insect moves its wings:


(2) $$s_{\mathrm{wing}\;\mathrm{beat}}(t)=\sum_{n=1}^{N}A_{n}\exp\;(2\pi nf_{w}t+\varphi_{n}),$$

where $A_{n}$ and $\varphi_{n}$ are, respectively, the effective amplitude and phase shift induced by the wing-beating at *n*th harmonic frequency (visualized on Fig. 3). Different insect species likely exhibit different patterns of wing movements (Fig. 4), which, as we later demonstrate, can be observed in the composition of the harmonic spectrum of $s_{\mathrm{wing}\;\mathrm{beat}}(t)$. The harmonic frequencies from the wing movement during flight are distinguishable on the spectrogram. We go on to explore this harmonic spectrum to train a ML model to identify and classify different flying insect species.

### Collection of micro-Doppler signatures

The transceiver structure is minimal, comprising a signal generator, signal coupler, antenna, and down-converter, as illustrated in Fig. 2. This design was followed for two reasons, namely: (i) to allow the collection of generalized data, with as few hardware-specific aspects as practicable for a radar system, in order to build generalized training labeled datasets for radar-based ML; and (ii) to keep the cost of the device as low as possible, to support large-scale deployment.

This configuration does not allow measurement of the exact location of the target. However, for our purposes, the exact location is irrelevant, so long as the target is in relative proximity (that is, within several cm) of the antenna, a functionality ensured by using low power. Detection, identification, and quantification of the relative abundance of individual species is the primary goal of the technology.

### Preprocessing of the signal

The signal is normalized to reduce the effects of noise. It is then high-pass filtered using a second-order Chebyshev high-pass filter with a cutoff frequency of 50 Hz to remove slow-moving background noise. This choice of filter frequency was based on the reported wing-beat frequency of hymenopteran pollinators, which is typically above 50 Hz (24).

Insect wing flapping is characterized by patterns of harmonics in the signal. This periodic structure of the signal can help in determining whether the signal segment has micro-Doppler features or not. To isolate such segments, the mmWave signal is first divided into overlapping frames using a Hann window of 2 s in length with a 1-s overlap. For each frame, the harmonic ratio is computed. The harmonic ratio provides a quantitative measure of the degree of periodicity (harmonicity) present in a signal. It reflects the relative strength of harmonic components with respect to the overall energy of the signal. This metric is computed using the normalized autocorrelation function, which compares a signal segment with time-lagged versions of itself (25). Given a signal frame *x* of length *L*, the normalized autocorrelation Ψ(k) is calculated for each lag *k*, and the harmonic ratio $\eta_{\mathrm{HR}}$ is defined as the maximum value of Ψ(k) within a specified lag interval $\left[K_{0},K\right]$, where the maximum lag is set to


$$K=0.04F_{s}=40\;\text{samples},$$

corresponding to 40 ms at $F_{s}=1$ kHz and a minimum fundamental frequency of 25 Hz. The lower bound $K_{0}$ is not chosen manually but is determined adaptively for each frame as the first zero-crossing of Ψ(k), ie the smallest k>0 such that


Ψ(k−1)Ψ(k)≤0.

Kim et al. (25). By construction, $\eta_{\mathrm{HR}}$ is constrained to the interval [0,1]. A higher harmonic ratio indicates a stronger periodic structure, making it a useful indicator for detecting micro-Doppler signatures associated with repetitive motion, such as insect wing flapping. Segments with a harmonic ratio exceeding a predefined threshold are considered to contain micro-Doppler features. They are retained for further analysis. In the example shown on Fig. 3, the insect (a common wasp, *Vespula vulgaris*) remained stationary for the first 6 s, during which the harmonic ratio remained below 0.4. At 6 s, the insect began hovering, and both the harmonic ratio and the spectrogram reveal the emergence of periodic micro-Doppler patterns. This behavioral change was confirmed by the corresponding video footage. The most obvious feature in Fig. 3(a) after 6 s is a slow increase in the mean amplitude (a very low-frequency component); the micro-Doppler wingbeat contribution instead appears as rapid, tiny modulations that make the trace appear thicker in this interval.

The same frame selection process is applied across the full duration of each insect’s flight recording. Therefore, the number of flight segments reported in Table 1 corresponds directly to the number of overlapping frames selected based on this harmonic thresholding approach. Additionally, the segmentation was repeated using shorter window lengths ranging from 1 s down to 0.1 s to generate additional segment sets, also summarized in Table 1.

### Micro-Doppler feature extraction

The study aims to capture species-specific patterns by extracting relevant micro-Doppler features from the micro-Doppler detected signal segment. Specifically, harmonic, spectral, and temporal features were all computed and evaluated as possible candidates for successful classification. Feature extraction was implemented in MATLAB using standard functions, including spectral features, cepstral analysis, PEFs, and Mel-frequency cepstral coefficients (MFCCs) (26). Custom scripts were developed to compute harmonic ratio and band-power features from the micro-Doppler spectra. All harmonic, spectral, and temporal features computed and evaluated during the feature extraction stage, comprising a total of 70 features, are summarized in Table S1.

### ML classification

We used a hybrid, multistage classification algorithm that leverages the complementary strengths of CatBoost (27) and ExtraTrees classifiers (28). This approach leverages the complementary capabilities of ensemble learning modeling, encapsulated in a structured process designed to enhance predictive accuracy and adaptability.

#### Model initialization and training

A micro-Doppler dataset was constructed by extracting relevant features from all micro-Doppler segments obtained from various insect species. For model initialization and training, the dataset was divided into training and test sets, with 80% allocated for training and 20% for testing. Care was taken to ensure that data from the same subject did not appear in both the training and test sets, thereby preventing potential data leakage. To address class imbalance, class weights were applied during training to penalize the errors in minority classes. Alternative approaches such as undersampling the majority class and oversampling the minority class (eg synthetic augmentation) were also explored, but these did not improve performance with the available dataset.

A hierarchical classification framework was implemented to predict insect species in a staged manner, reflecting the natural taxonomic structure of the data. CatBoost was used in the initial stages to discriminate between broader insect groups, as gradient boosting effectively captures high-level nonlinear relationships and achieves low bias through iterative error correction. In the final stage, where classes exhibit highly similar micro-Doppler signatures and separability is driven by subtle spectral and temporal variations, an ExtraTrees classifier was employed, as its randomized split selection and ensemble averaging can reduce variance and provide more robust generalization under limited class separability. In the first stage, a CatBoost classifier was trained to distinguish between the families Vespidae and Apidae, using all available training samples. Instances classified as Vespidae at this stage were directly assigned the species *V. vulgaris*, as no further subclass differentiation was required. Samples classified as Apidae proceeded to the second stage, where a separate CatBoost model predicted the genera *Apis*, and *Bombus*. Predictions of *Apis* were mapped directly to the species *A. mellifera*, while samples identified as *Bombus* were passed to a third-stage classifier. This final model, implemented using an ExtraTrees classifier, was trained to discriminate between the three *Bombus* species (*B. lapidarius*, *B. muscorum*, and *B. terrestris*).

#### Model testing

The trained hierarchical classifiers were evaluated on the test set, which contained samples from individuals not seen during training. At each stage of the hierarchy, predictions were generated and passed to the subsequent stage if required. Overall classification performance was assessed at the species level by comparing the final predicted labels with the true labels.

In multiclass classification problems, particularly when there is significant variation in class distributions, the F1-score is a more appropriate metric to assess the model’s effectiveness (29). Thus, the F1-score is used:


(3) $$\text{F1}=\frac{2\times \text{Precision}\times \text{Recall}}{\text{Precision}+\text{Recall}},$$

where $\text{Precision}=\frac{\mathrm{TP}}{\mathrm{TP}+\mathrm{FP}}$ and $\text{Recall}=\frac{\mathrm{TP}}{\mathrm{TP}+\mathrm{FN}}$. Here, TP (true positives) denotes correctly classified positive instances, FP (false positives) denotes instances incorrectly classified as positive, and FN (false negatives) denotes positive instances incorrectly classified as negative. Accuracy is defined as the proportion of correctly classified samples, computed as the sum of the diagonal elements of the confusion matrix divided by the total number of samples. In a multiclass single-label classification setting, this quantity is numerically equivalent to the microaveraged F1 score, since each sample contributes exactly one predicted label and one ground-truth label. In addition, precision, recall, and F1 scores were reported individually for each species to provide class-level evaluation under imbalance. Confusion matrices were generated to visualize misclassification patterns and report cumulative end-to-end predictions at each stage, retaining classes finalized in earlier stages even though they are excluded from subsequent training.

In addition, feature importance values were extracted using SHAP, providing insight into which harmonic, spectral, and temporal features contributed most strongly to classification decisions.

## Results

### Pollinator insect species classification

**Figure 5:**
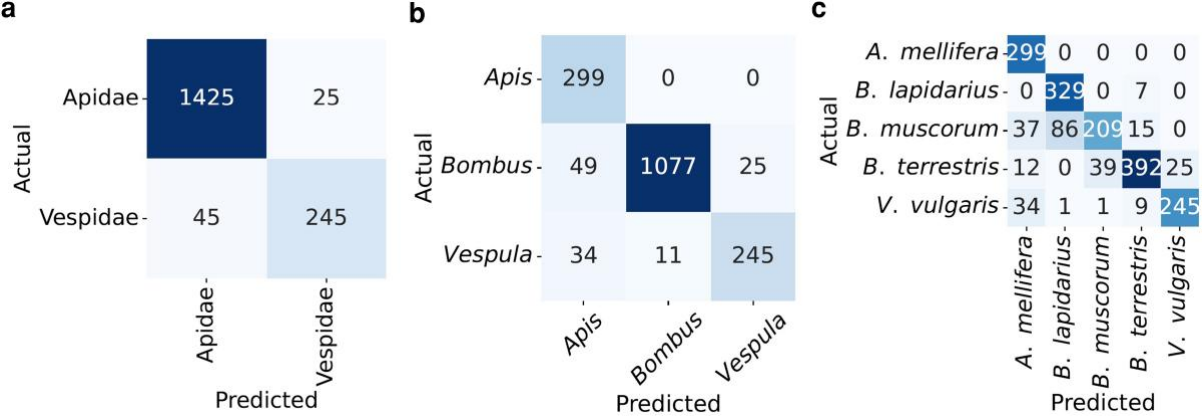
Confusion matrices showing the cumulative classification outcomes of the hierarchical framework: a) first-level family classification distinguishing Vespidae and Apidae; b) second-level genus classification differentiating *Apis* and *Bombus*, with *Vespula* retained from the first level; and c) final species-level classification identifying *Vespula vulgaris*, *Apis mellifera*, *Bombus lapidarius*, *Bombus muscorum*, and *Bombus terrestris*.

**Figure 6:**
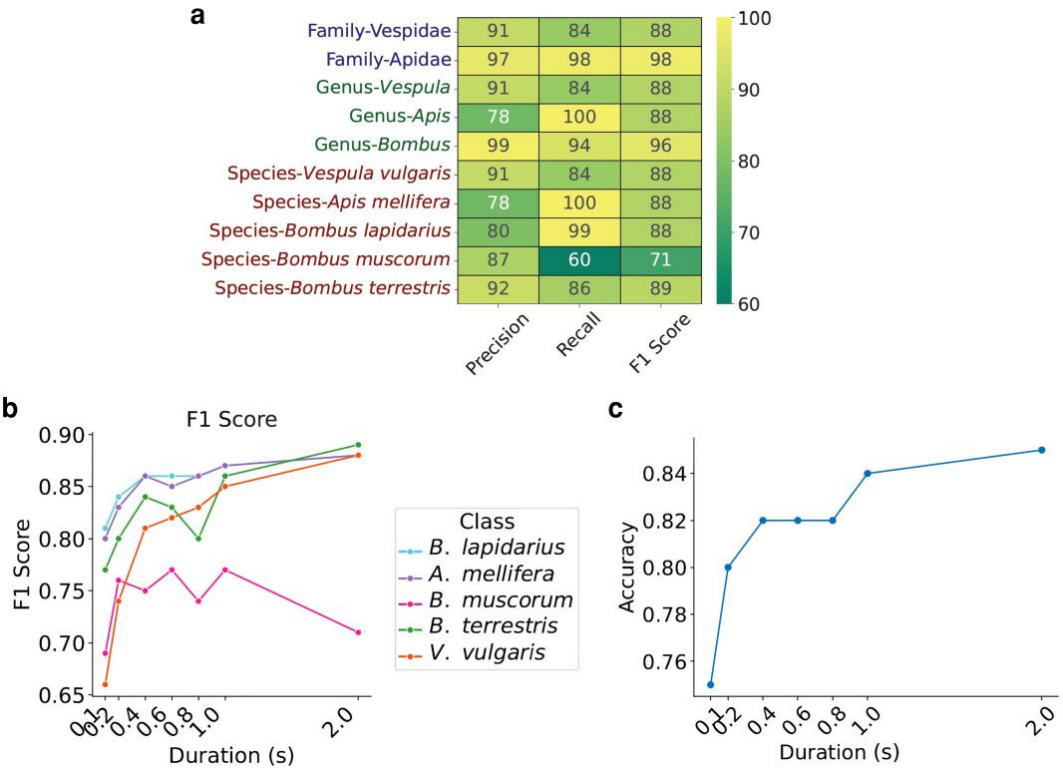
Performance of the hierarchical classification model at different taxonomic levels. a) Hierarchical performance metrics (precision, recall, and the F1 score), heatmap across various categories. Precision indicates the accuracy of positive predictions, recall shows the model’s ability to find all relevant instances and the F1 Score represents the balance between precision and recall. b) Changes in the F1 score with airborne signal duration (0.1–2 s) for our five focal species (*Vespula vulgaris*, *Apis mellifera*, *Bombus lapidarius*, *Bombus muscorum*, and *Bombus terrestris*). c) Variation in overall classification accuracy with signal duration.

At the family level, the first-stage classifier distinguished between the Apidae and Vespidae families with an accuracy of 96% (Fig. 5a). Additional performance metrics, including precision, recall, and F1 score (a measure of the balance between precision and recall; see Methods), further indicate robust classification performance (Fig. 6a). Samples classified as Vespidae at this stage were directly assigned to *V. vulgaris*.

Samples classified as Apidae were passed to a second-stage classifier that differentiated between the genera *Apis* and *Bombus*. Predictions finalized in the previous stage (*V. vulgaris*) were retained unchanged, and the cumulative end-to-end classification accuracy at this stage was 93% (Fig. 5b). Samples classified as *Apis* were directly mapped to *A. mellifera*, while those classified as *Bombus* were forwarded to the third-stage model.

The third-stage classifier operated only on samples identified as *Bombus* and discriminated among the species *Bombus lapidarius*, *B. muscorum*, and *B. terrestris*. Predictions for *A. mellifera* and *V. vulgaris*, finalized in earlier stages, were carried forward unchanged. Considering all species jointly, the cumulative species-level classification accuracy was 85% (Fig. 5c). Notably, the F1 scores for these species were comparable, except for *B. muscorum*, which showed lower values likely due to smaller sample size (Table 1).

### Duration of recorded wing flapping

In order to capture relevant temporal features unique to each insect species, the algorithm analyzes fragments of varying duration. Longer durations generally improve classification accuracy but are harder to record because insects must remain within the antenna range for the entire interval. Figure 6b presents F1 scores across signal durations of 0.1, 0.2, 0.4, 0.6, 0.8, 1, and 2 s for the five species considered. The number of samples available for each species and duration is given in Table 1. Each segment was used only once, at the maximum available duration, to prevent overlap across durations. Durations longer than 2 s were explored by applying longer analysis windows to the raw flight recordings, but insufficient valid samples were available for statistical evaluation. Durations shorter than 0.1 s were not considered, since at such brief intervals frequency resolution becomes too coarse to capture wingbeat harmonics reliably. F1 score improvement with increasing signal duration is consistent across all categories except in *Bombus muscorum*. We find that, as the duration of airborne signals increase, the model’s ability to classify them accurately also improves (Fig. 6c), as would be intutively expected. When using micro-Doppler signal segments of 2 s, classification accuracy reached 85%. However, as wing-flapping duration decreased, species classification accuracy also declined, with overall accuracy dropping to 75% at a signal duration of 0.1 s (Fig. 6c).

### Feature importance analysis

**Figure 7:**
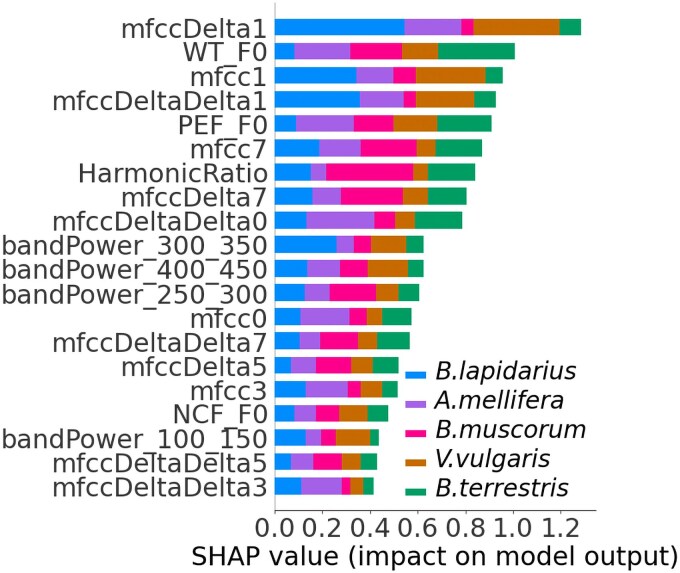
Features importance and their contribution to prediction evaluation using SHAP, showing the top 20 features influencing species classification.

Figure 7 illustrates the influence of key features of the micro-Doppler spectrum on species classification, as explained by SHAP (30). SHAP values quantify the impact of each feature on the model’s output, where higher SHAP values indicate greater importance for the species classification task. Features with larger SHAP values, such as mfccDelta1 and WT_F0, have a stronger influence on the classification, while lower SHAP values signify features with lower influence.

The top-ranked feature, mfccDelta1 (first-order delta Mel-Frequency Cepstral Coefficient), represents the rate of change in the spectral characteristics of the reflected signal, capturing the temporal dynamics of the insect wing beats through changes in the wing-beat-induced spectral envelope, rather than the wing-beat frequency itself. This feature plays a particularly significant role in classifying multiple species, with notable contributions to the prediction of *Bombus lapidarius* and *Vespula vulgaris* (Fig. 7). WT_F0, the second most important feature, reflects the fundamental wing beat frequency as determined by wavelet transform analysis. It is especially influential for classifying *Bombus terrestris* and *Bombus muscorum* (Fig. 7). In the case of *Apis mellifera*, the primary influential feature is PEF_F0, representing the fundamental frequency extracted using a PEF.

The top 20 features include both first-order and second-order delta coefficients of the MFCCs, which capture dynamic characteristics of the insect wing beat patterns from the reflected signal. Specifically, the first-order delta coefficients (or delta MFCCs) such as mfccDelta1, mfccDelta7, and mfccDelta5 represent the rate of change in the spectral characteristics of the reflected signal, providing insight into how quickly the insect wing movements evolve. These features help differentiate species with rapidly fluctuating wing beats from those with more stable patterns.

Second-order delta coefficients (delta–delta MFCCs), including mfccDeltaDelta0, mfccDeltaDelta1, mfccDeltaDelta3, mfccDeltaDelta7, and mfccDeltaDelta5, capture the acceleration of these changes, offering information about the smoothness or abruptness of wing movement transitions across time. These features help differentiate species with more erratic or irregular wing motion from those with smoother, consistent beats. Additionally, individual MFCCs such as mfcc0, mfcc1, mfcc3, and mfcc7 capture the overall spectral characteristics such as the energy distribution and the spectral shape of the reflected signal from the insect wing beats. Figure S3 shows representative examples of MFCC and their derivatives for flight recordings.

The fundamental frequencies (F0) calculated using the wavelet transform (WT_F0) and PEF (PEF_F0) are indicative of the primary wing beat frequency of the insect. As species vary in their wing beat rates due to differences in wing size and flight behavior, these features help distinguish species with higher wing beat frequencies, which exhibit higher F0 values, from slower-flapping species, which exhibit lower F0 values.

Band power ratios, such as those within the frequency ranges of 300 to 350 Hz (bandPower_300_350), 400 to 450 Hz (bandPower_400_450), 250 to 300 Hz (bandPower_250 _300), and 100 to 150 Hz (bandPower_100_150), reflect how the energy of the reflected signal is distributed across specific frequency bands. These ratios help identify species-specific wing beat signatures, as different species may exhibit distinct energy distributions due to variations in wing morphology and movement. Finally, the harmonic ratio of the signal measures the presence of periodic, harmonic patterns in the reflected signal, which arise from the regularity of wing flapping. A higher harmonic ratio suggests a more structured, periodic wing movement, which can be characteristic of certain species, while a lower harmonic ratio could indicate more complex wing flapping behaviors.

We found distinct separation in micro-Doppler feature distributions among species (Figure S2), indicating significant clear interspecific variation, demonstrating that these features underpin the reliable classification of species with minimal overlap.

## Discussion

This work presents an alternative solution that successfully analyzes the micro-Doppler spectrum originating from wing movements, offering sufficient data for robust species-level classification. Although our models misclassified some individuals at the species level, our results are, nonetheless, highly promising, and demonstrate that the system can reliably identify insects at more coarse taxonomic levels, such as genus or family, suggesting robust capabilities for broader-level identification. Though we focused on the Hymenoptera due to their role as critical pollinators in ecosystems, our proposed approach can easily be adapted to track pests and invasive insect species from other groups.

We found that longer wing-flapping durations significantly improved classification accuracy. For instance, using a 1 s micro-Doppler segment, the species-level accuracy in our models reached 84%, compared to 75% for a 0.1 s segment. This highlights the need to maintain target insects within the beam of the radar system for as long as practicable. Future implementations for field applications may consider a trap-like structure as suggested in previous work (31), that guides the insect to follow a certain pattern while being analyzed by the system, with the insect being released unharmed once the analysis is complete.

Among the various features extracted, top features identified through SHAP analysis indicate that species classification is supported not only by wingbeat frequency but also by other key spectral characteristics, such as cepstral coefficients and bandpower across different frequency bins. Some of the MFCC-derived features that emerged as useful for species classification may, in part, be influenced by the restricted flight conditions of our experimental setup. Because the insects were confined to a relatively small enclosure, occasional contact with the walls could have introduced short-term variations in the radar echoes. Although frequency-domain features are the primary focus of this study, the signal-to-noise ratio (SNR) is another factor that may vary in real-world deployments as insects fly toward or away from the radar. In our controlled experiments, the insects were constrained to a limited volume, which minimized such range-dependent effects, but in free-flight conditions the robustness of extracted features may be impacted by variable SNR. The engineered feature set used here is deliberately broad and includes correlated quantities describe similar aspects. Although the tree-based models can down-weight less informative variables, a dedicated feature-elimination or feature-selection study to derive a more compact, nonredundant subset of micro-Doppler features would be a useful direction for future work, particularly for deployment on resource-constrained platforms.

Although the SHAP ranked top features namely MFCC-based descriptors (mfccDelta1, mfcc1, mfccDeltaDelta1, mfcc7, mfcc0, mfcc3, mfccDelta7, mfccDelta5, mfccDeltaDelta0, mfccDeltaDelta5, and mfccDeltaDelta3), wingbeat frequency estimates (WT_F0, PEF_F0, NCF_F0), the harmonic ratio (HarmonicRatio), and bandpower features in the 100–150, 250–300, 300–350, and 400–450 Hz bands (bandPower_100_150, bandPower_250_300, bandPower_300_350, and bandPower_400_450) enhanced discrimination among species in this controlled setting, their relevance under free and natural flight conditions remains uncertain. This limitation highlights the “proof-of-concept” nature of our study. Future developments of this approach should focus on field trials with free-flying insects in natural habitats, enabling the capture of longer flight trajectories under realistic conditions.

As with any ML solution, the proposed system is critically reliant on the training database, that is, the large collection of recorded radar signatures that are labeled with ground truth information. Contrary to visual images, such databases do not yet exist. While this study demonstrated classification for five key species, there is potential for significantly increasing this number as more mmWave signatures, including micro-Doppler spectra of various species, are recorded and shared publicly. Furthermore, as this approach relies on a data-driven model rather than species-specific mathematical models, it is adaptable and scalable, allowing it to classify a wide variety of insect species without the need for specialized models. Ultimately, our goal is to create a global database of insect radar signatures, enabling the instantaneous classification of any insect within the system’s range. To strengthen the robustness of this approach, future work will incorporate more extensive evaluation strategies such as cross-validation and repeated trials, as well as methods to mitigate class imbalance, particularly for underrepresented species. In practical deployments, species-rich communities will require larger datasets and more complex hierarchical models spanning families, genera, and species. The present framework is readily extensible, and future work will focus on expanding the taxonomic scope to make the system applicable to natural insect communities. In future versions of the insect radar signature database, environmental metadata will be systematically recorded alongside the radar signals. In particular, ambient temperature will be included, as it directly influences wingbeat frequency and other flight-related parameters (32, 33). Relative humidity will also be captured to provide additional context, enabling subsequent studies to investigate its potential role in shaping insect behavior and radar signatures.

Overall, our method offers a nonlethal, fast, and cost-efficient solution to monitor flying insects. Looking ahead, the framework can be integrated into emerging mmWave communication and sensing infrastructures (eg 5G/6G and IoT), enabling scalable, networked, and continuous biodiversity monitoring by leveraging existing hardware for distributed sensing and real-time data transfer. Future work may push the limit of the technology to provide not only species classifications and detection of changes in biodiversity and community structure but also potentially to monitor shifts in insect behavior through detection of unusual perturbations in their wing-beat patterns.

## Supplementary Material

pgag096_Supplementary_Data

## Data Availability

Data used in the study are available at Permalink: https://ieee-dataport.org//documents/raw-mmwave-radar-dataset-pollinating-insects-micro-doppler-based-classification-and DOI: dx.doi.org/10.21227/8397-ch84 (34).
