# Supplementary material for: Harnessing mmWave signals and machine learning for noninvasive taxonomic classification of insects
Source: PNAS Nexus. 2026 Apr 28;5(4):pgag096. doi: 10.1093/pnasnexus/pgag096 (PMC13122614; doi:10.1093/pnasnexus/pgag096)
Supplement: pgag096_Supplementary_Data [file pgag096_supplementary_data.pdf]

Supplementary Information: Harnessing mmWave Signals and Machine Learning for Non-Invasive Taxonomic Classification of Insects

Linta Antony[a], Cian White[a], Nicola Marchetti[a], Ian Donohue[a], Jane C. Stout[a], Adam Narbudowicz[b,*]

[a] Trinity College Dublin, Ireland

[b] Technical University of Denmark, Kgs. Lyngby, Denmark

* Corresponding author: adana@dtu.dk

Insect biodiversity | Species recognition | Machine learning | Micro-Doppler signatures | mmWave | radar | Hierarchical classification

Target detection range evaluation

Time–frequency analysis using the STFT was employed to analyse the signals received at different target distances. Taken together, the STFT at 0 cm shown in Fig.4b and the distance-dependent STFTs in (Fig. S1) reveal clear and distinct spectral patterns across all measured ranges, indicating that the wingbeat-induced harmonic structure remains observable from the 0 cm configuration up to the maximum experimentally evaluated distance of 18 cm.

A theoretical estimate of the maximum detectable range of the proposed radar system was obtained using the monostatic radar range equation

$$R = \left(\frac{P_t G^2 \lambda^2 \sigma}{P_r (4\pi)^3} \right)^{1/4}, \tag{1}$$

where P_t and P_r denote the transmitted and received powers, respectively, G is the antenna gain, λ is the wavelength, and σ is the radar cross section (RCS) of the target.

For the experimental configuration, the transmitted power was set to 20 dBm (100 mW) and the antenna gain was 19.92 dB. A target positioned directly at the antenna aperture (0 cm) produced a received signal power of approximately -70 dBm (10^{-10} W), which was used as a estimate of the minimum detectable received power. The operating wavelength was 1 cm, corresponding to a carrier frequency of 30 GHz. Recent Ku-band work on migratory insects reports equivalent RCS values of about 0.002–0.005 cm^2 for insects of similar size [1]. Consistent with these results, we adopt an effective RCS of 0.002 cm^2 as a conservative value for a bee-sized insect in our range estimate. Substituting this RCS, together with the measured transmit power, receive threshold, antenna gain and wavelength, into the monostatic radar range equation yields a theoretical detection range of approximately 55.8 cm, which we interpret as a conservative, order-of-magnitude estimate for the current hardware configuration.

This value represents an idealised upper-bound estimate under free-space assumptions and does not imply reliable experimental detection at this distance.

The range trials were conducted for a single species and were intended to illustrate the detectability of micro-Doppler harmonics at varying distances rather than to establish an empirical maximum detection range or evaluate classification accuracy. Under the present measurement conditions, reliable micro-Doppler signatures were experimentally observed up to 18 cm.

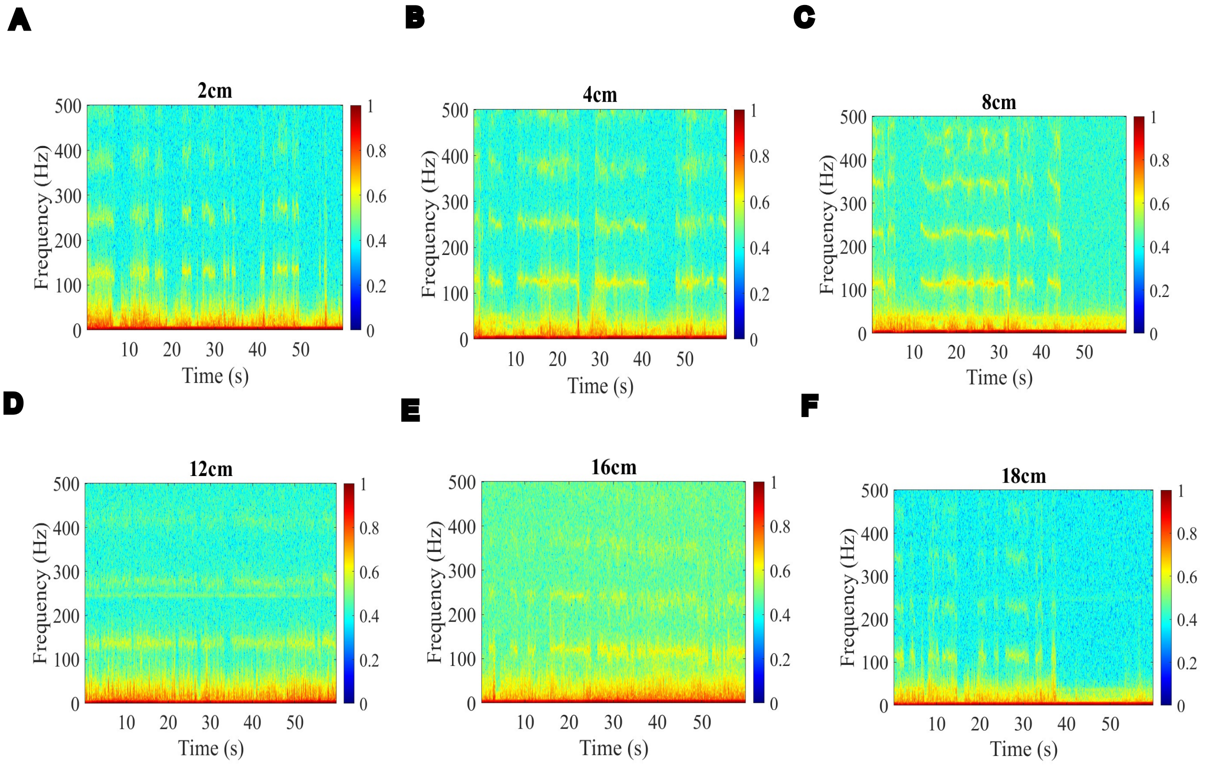

Figure S1: Time–frequency analysis using STFT on unfiltered reflected signals from wing flapping *Bombus terrestris* obtained at distances ranging between 2 and 18 cm from the antenna.

Supplementary References

References

[1] Rui Wang et al. “Insect-equivalent radar cross-section model based on field experimental results of body length and orientation extraction”. In: *Remote Sensing* 14.3 (2022), p. 508.

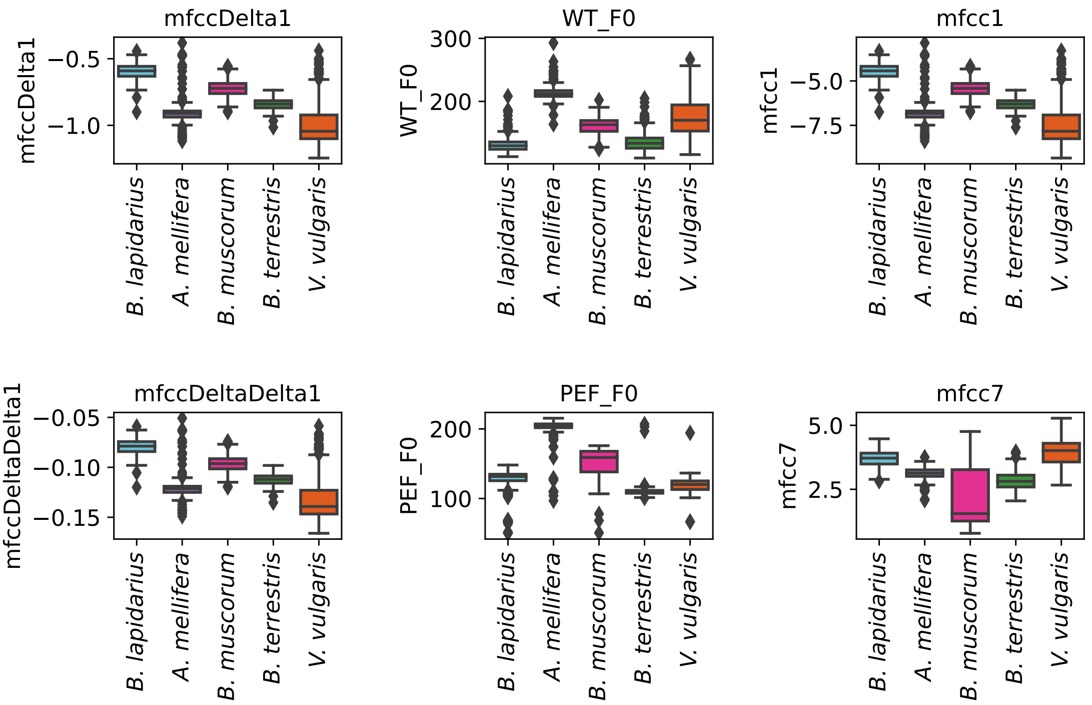

Figure S2: Box plots of the six features that contributed most to the classification of our focal insect species (see Fig.7).

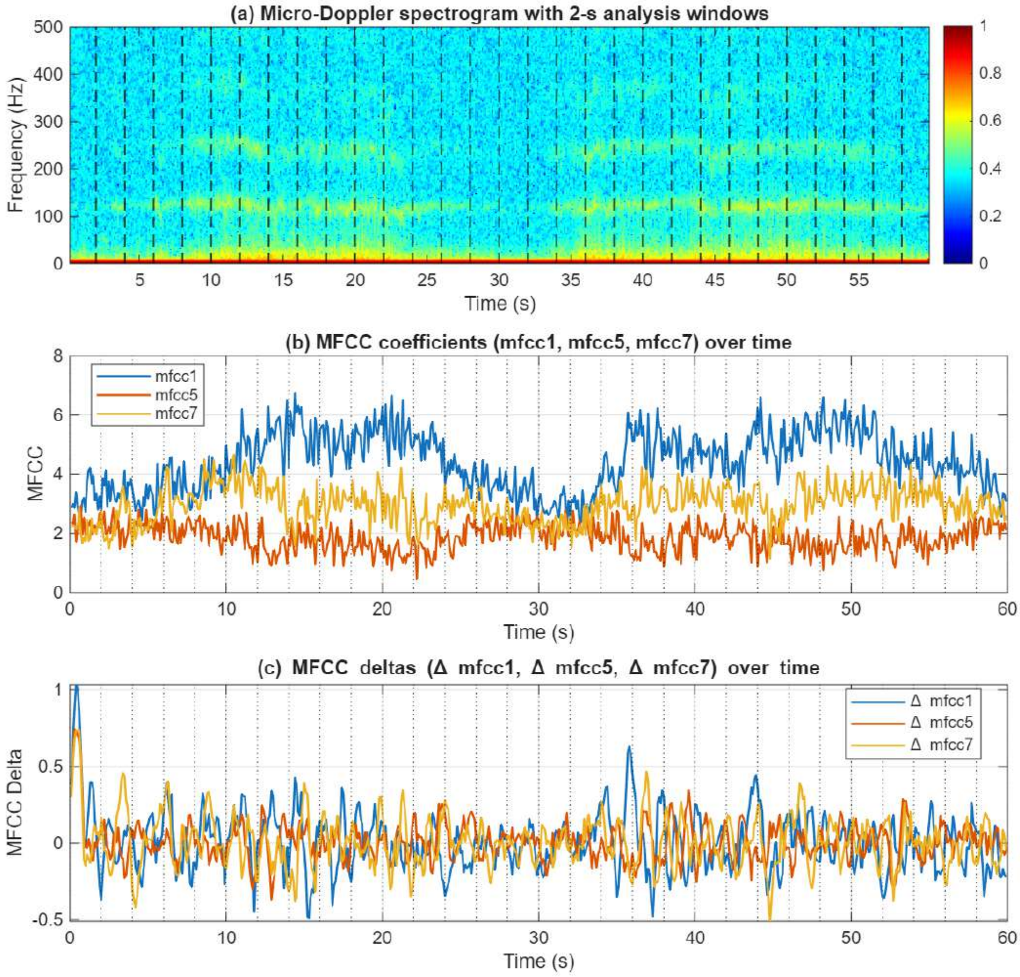

Figure S3: Illustrative relationship between micro-Doppler structure and MFCC-based features, which contributed most to the classification of our focal insect species (see Fig.7) (a) Micro-Doppler spectrogram of the unfiltered radar return over a 60 s interval, with vertical dashed lines indicating consecutive 2 s analysis windows used in the classification pipeline. (b) Frame-level MFCC coefficients (`mfcc1`, `mfcc5`, `mfcc7`), capturing slow variations in the spectral envelope of the reflected signal. (c) Corresponding first-order MFCC derivatives (Δ`mfcc1`, Δ`mfcc5`, Δ`mfcc7`), which quantify the short-term rate of change of the spectral envelope. The 60 s duration is shown for visualisation only; all classification are performed exclusively on 2 s segments.

Table S1: Detailed description of extracted harmonic, spectral, and temporal features

Feature Type	Feature Name (Variable)	Explanation
**Harmonic Features**	Harmonic Ratio (`HarmonicRatio`)	Proportion of energy in harmonic components relative to total signal energy.
	STFT Fundamental Frequency (`STFT_F0`)	Fundamental frequency identified from using a Welch-type power spectrum, with 39 % of the available samples applied per segment .
	Harmonics, 1st–5th Order (`Harmonics_1-5`)	Harmonics identified as multiples of the fundamental frequency from STFT peaks.
	Pitch Estimation Filter Fundamental Frequency (`PEF_F0`) [**gonzalez2011pitch**]	Fundamental frequency estimated using a pitch estimation filter.
	Cepstrum-based Fundamental Frequency (`CEP_F0`)[**noll1967cepstrum**]	Fundamental frequency derived via cepstral analysis.
	Wavelet Transform Fundamental Frequency (`WT_F0`)	Fundamental frequency obtained through wavelet-based analysis.
	Normalized Correlation Fundamental Frequency (`NCF_F`)[**atal1972automatic**]	Fundamental frequency identified by maximizing normalized correlation function.
**Spectral Features**	Spectral Centroid (`SpectralCentroids`)	Center of mass of the frequency spectrum, indicating perceived brightness.
	Spectral Flatness (`SpectralFlatness`)	Measure of how tonal or noise-like the signal is (Wiener entropy).
	Spectral Bandwidth (`SpectralBW`)	Width of the frequency spectrum around its centroid.
	Spectral Roll-off (`SpectralRollOff`)	Frequency below which 95% of spectral energy is contained.
	Spectral Flux (`SpectralFlux`)	Rate of change in the power spectrum between successive frames.
	Spectral Skewness (`SkewnessValue`)	Asymmetry of frequency distribution around its mean frequency.
	Spectral Kurtosis (`KurtosisValue`)	Measure of peakedness or sharpness of frequency distribution.
	Spectral Decrease (`SpectralDecrease`)	Describes how spectral energy declines from lower to higher frequencies.
	Mel-Frequency Cepstral Coefficients (`mfcc`, `mfccDelta`, `mfccDeltaDelta`)	Cepstral representation of the short-term power spectrum of the signal based on perceptual (mel) frequency scaling. MFCCs are extracted as a vector of cepstral coefficients (indexed as `mfcc0`, `mfcc1`, . . .), representing the spectral envelope of the reflected signal. First-order temporal derivatives (`mfccDelta`) are computed for each MFCC coefficient and capture the rate of change of the spectral envelope over time, while second-order derivatives (`mfccDeltaDelta`) capture the acceleration of these changes, reflecting the smoothness or abruptness of temporal spectral evolution associated with insect wing-beat motion.
	Band Power Ratio (`BPR_0_50Hz`, etc.)	Ratio of power within each specified frequency band (0–50 Hz, 50–100 Hz, 100–150 Hz, 150–200 Hz, 200–250 Hz, 250–300 Hz, 300–350 Hz, 350–400 Hz, 400–450 Hz, and 450–500 Hz) relative to total spectral power.
**Temporal Features**	Zero Crossing Rate (`zcr`)	Number of times the high-pass filtered signal changes sign per unit time, reflecting its oscillatory frequency content
	Root Mean Square Energy (`rmsEnergy`)	Overall signal energy level in the frame.

Although multiple methods are included for estimating the fundamental frequency, these provide complementary perspectives on the same parameter. Their relative usefulness is later assessed through feature importance analysis, which identifies the most informative representation for classification
